# Supplementary material for: Transcriptome Profiling of Cardiac Glycoside Treatment Reveals EGR1 and Downstream Proteins of MAPK/ERK Signaling Pathway in Human Breast Cancer Cells
Source: Int J Mol Sci. 2023 Nov 2;24(21):15922. doi: 10.3390/ijms242115922 (PMC10647710; doi:10.3390/ijms242115922)
Supplement: Supplementary file 1 [file ijms-24-15922-s001.zip › Figure S1. Functional_revised_legend.pdf]

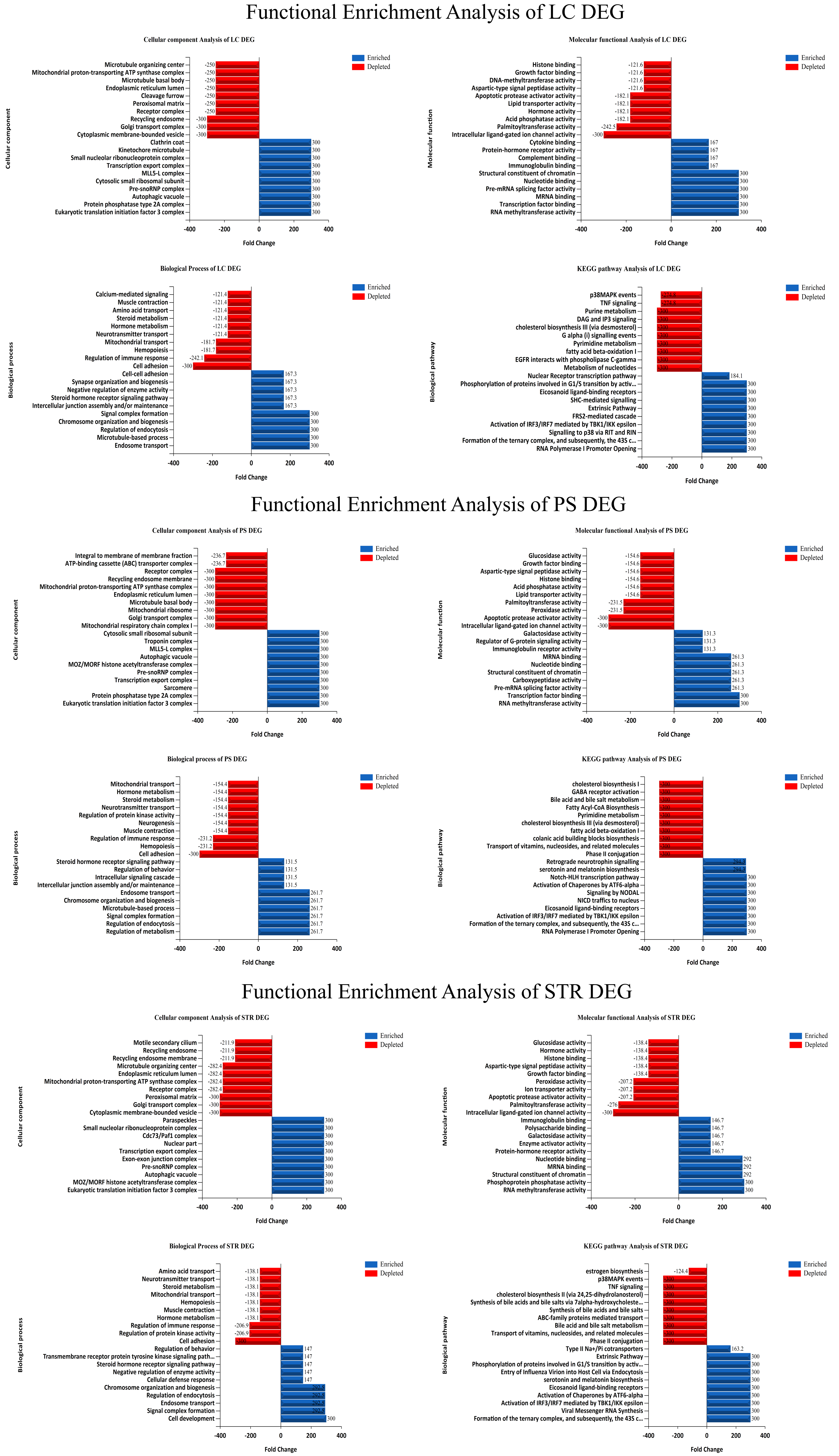

Figure S1. Functional enrichment analysis of differentially expressed genes on LC,PS, and STR treatment. The analysis covered cellular components, molecular function, biological process,and KEGG pathway delineating the crucial genes involved in the anticancer activity of the compounds.
