# Supplementary material for: Transcriptome Profiling of Cardiac Glycoside Treatment Reveals EGR1 and Downstream Proteins of MAPK/ERK Signaling Pathway in Human Breast Cancer Cells
Source: Int J Mol Sci. 2023 Nov 2;24(21):15922. doi: 10.3390/ijms242115922 (PMC10647710; doi:10.3390/ijms242115922)
Supplement: Supplementary file 1 [file ijms-24-15922-s001.zip › Figure S2. Docking_revised_legend.pdf]

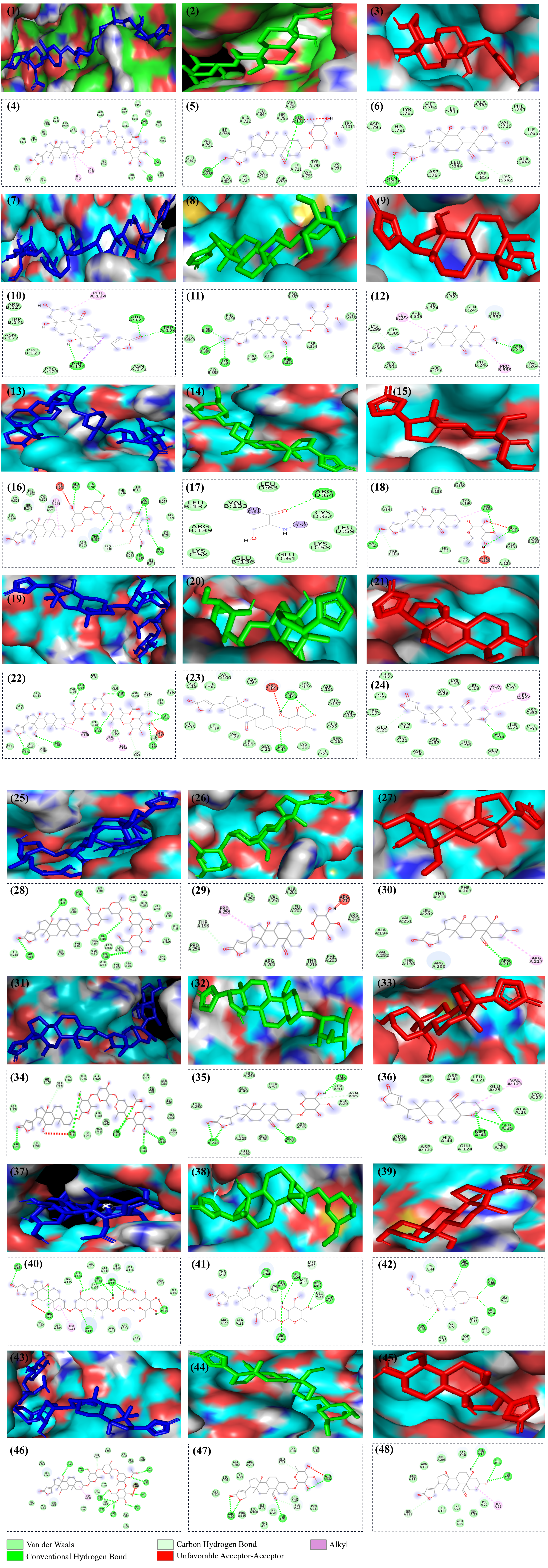

Figure S2. Molecular docking interactions of LC, PS, and STR ligands. (1-6) Illustrates the interactions of ligand molecules with p53 protein in 3D and 2D images. (7-12) Illustrates the interactions of ligand molecules with CCNK protein in 3D and 2D images. (13-18) Illustrates the interactions of ligand molecules with CASP9 protein in 3D and 2D images. (19-24) Illustrates the interactions of ligand molecules with BCL2L1 protein in 3D and 2D images. (25-30) Illustrates the interactions of ligand molecules with CDK7 protein in 3D and 2D images. (31-36) Illustrates the interactions of ligand molecules with CDK2 protein in 3D and 2D images. (37-42) Illustrates the interactions of ligand molecules with CDK2AP1 protein in 3D and 2D images. (37-42) Illustrates the interactions of ligand molecules with CDKN1A protein in 3D and 2D images.
