# Supplementary material for: Transcriptome Profiling of Cardiac Glycoside Treatment Reveals EGR1 and Downstream Proteins of MAPK/ERK Signaling Pathway in Human Breast Cancer Cells
Source: Int J Mol Sci. 2023 Nov 2;24(21):15922. doi: 10.3390/ijms242115922 (PMC10647710; doi:10.3390/ijms242115922)
Supplement: Supplementary file 1 [file ijms-24-15922-s001.zip › Table S1.pdf]

Table S1. Primer sequence of used for qRT-PCR validation of selected genes.

Gene	Forward Sequence	Reverse Sequence
EGR1	GAGAAGGTGCTGGTGGAGAC	TGGGTTGGTCATGCTCACTA
MAPK1	TGGATTCCCTGGTTCTCTCTAAAG	GGGTCTGTTTTCCGAGGATGA
CCNK	AGTGATGTATCTCGCAGGACG	ACAACCACGGCTCGCTTA
CASP9	CCTGCTTAGAGGACACAGGC	TGGTCTGAGAACCTCTGGCT
BCL2L1	AGGTCTTTTTCCGAGTGGCAGC	GCGTCCCAAAGTAGGAGAGGAG
CDK7	GGCACACCAACTGAGGAACA	AGTCGTCTCCTGCTGCACTG
CDK2	ATGGATGCCTCTGCTCTCACTG	CCCGATGAGAATGGCAGAAAGC
CDKN1A	AGGGGACAGCAGAGGAAG	GCGTTTGGAGTGGTAGAAATCTG
CDK2AP1	AGCATGGCAACGTC TTCACAGT	TGGCATTCCGTTCCGTTTCT
P53	TGCGTGTGGAGTATTTGGATGAC	CAGTGTGATGATGGTGAGGATGG
β-Actin	TGGCACCCAGCACAATGAA	CTAAGTCATAGTCCGCCTAGAAGCA
